# Myotonic dystrophy presenting as severely dilated cardiomyopathy with out-of-hospital cardiac arrest

**DOI:** 10.1007/s12471-018-1207-0

**Published:** 2018-11-27

**Authors:** M. Isrie, L. Wong, J. M. van Hagen, A. C. Houweling

**Affiliations:** 1 Department of Clinical Genetics, VU University Medical Centre, Amsterdam, The Netherlands; 2 Department of Cardiology, VU University Medical Centre, Amsterdam, The Netherlands

**Fig. 1:**
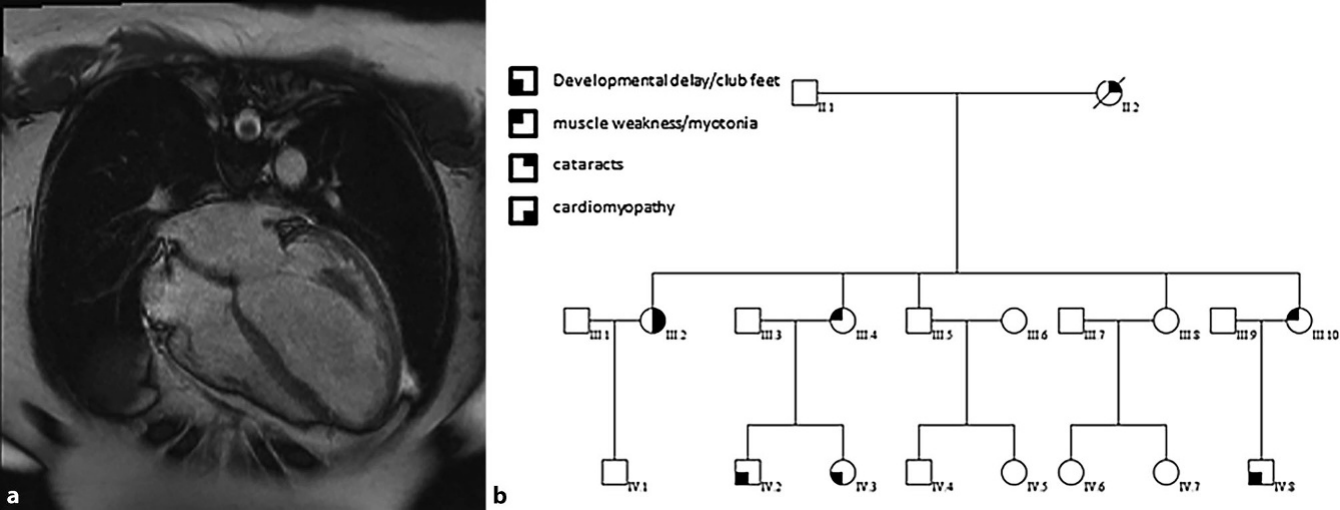
Cardiac MRI (**a**) and pedigree (**b**)

A 52-year-old female was admitted after an out-of-hospital cardiac arrest due to ventricular fibrillation. Cardiological evaluation revealed non-ischaemic dilated cardiomyopathy (DCM). Cardiac MRI showed a severely dilated left ventricle with an ejection fraction of 17% (Fig. 1a). Normal coronary arteries were seen on the coronary angiogram. Her medical history revealed cataracts at the age of 48 years and diminished strength in her hands. Two of her sisters had been diagnosed with myotonic dystrophy (MD). Their children had a more severe phenotype including clubfeet and developmental delay (Fig. 1b). Analysis of 53 cardiomyopathy-related genes using next-generation sequencing did not reveal any pathogenic variants. Analysis of the (CTG)*n* repeat in the *DMPK* gene (*n* > 150) revealed a heterozygous expansion, confirming the diagnosis of MD in our patient. A CTG repeat length between 100 and 1,000 is associated with the classic type of MD with muscle weakness and wasting, myotonia, cataracts and cardiac conduction abnormalities. DCM and ventricular fibrillation are previously reported but rare features of MD [1–5].
